# A New Approach in Detectability of Microcalcifications in the Placenta during Pregnancy Using Textural Features and K-Nearest Neighbors Algorithm

**DOI:** 10.3390/jimaging8030081

**Published:** 2022-03-19

**Authors:** Mihaela Miron, Simona Moldovanu, Bogdan Ioan Ștefănescu, Mihai Culea, Sorin Marius Pavel, Anisia Luiza Culea-Florescu

**Affiliations:** 1 Department of Computer Science and Information Technology, Faculty of Automation, Computers, Electrical Engineering and Electronics, Dunarea de Jos University of Galati, 47 Domneasca Str., 800008 Galati, Romania; mihaela.miron@ugal.ro (M.M.); simona.moldovanu@ugal.ro (S.M.); mihai.culea@ugal.ro (M.C.); 2 Department of Clinical Surgical, Faculty of Medicine and Pharmacy, Dunarea de Jos University of Galati, 47 Domneasca Str., 800008 Galati, Romania; 3 Department of Obstetrics and Gynecology, Clinical Emergency Hospital “Sf. Ap. Andrei” Galați, 800578 Galati, Romania; 4 Department of Electronics and Telecommunications, Faculty of Automation, Computers, Electrical Engineering and Electronics, Dunarea de Jos University of Galati, 47 Domneasca Str., 800008 Galati, Romania; sorin.pavel@ugal.ro

**Keywords:** placenta, calcifications, preterm placental calcifications, first order feature, second order feature, t-test, K-Nearest Neighbors algorithm, support vector machine algorithm

## Abstract

(1) Background: Ultrasonography is the main method used during pregnancy to assess the fetal growth, amniotic fluid, umbilical cord and placenta. The placenta’s structure suffers dynamic modifications throughout the whole pregnancy and many of these changes, in which placental microcalcifications are by far the most prominent, are related to the process of aging and maturation and have no effect on fetal wellbeing. However, when placental microcalcifications are noticed earlier during pregnancy, they could suggest a major placental dysfunction with serious consequences for the fetus and mother. For better detectability of microcalcifications, we propose a new approach based on improving the clarity of details and the analysis of the placental structure using first and second order statistics, and fractal dimension. (2) Methods: The methodology is based on four stages: (i) cropping the region of interest and preprocessing steps; (ii) feature extraction, first order—standard deviation (SD), skewness (SK) and kurtosis (KR)—and second order—contrast (C), homogeneity (H), correlation (CR), energy (E) and entropy (EN)—are computed from a gray level co-occurrence matrix (GLCM) and fractal dimension (FD); (iii) statistical analysis (t-test); (iv) classification with the K-Nearest Neighbors algorithm (K-NN algorithm) and performance comparison with results from the support vector machine algorithm (SVM algorithm). (3) Results: Experimental results obtained from real clinical data show an improvement in the detectability and visibility of placental microcalcifications.

## 1. Introduction

In recent years, digital image processing has been increasingly used in medical imaging where it has come to play a key role in the assisted diagnosis, planning, monitoring and evaluation of the treatment. Because medical images are complex, and due to their specific acquisition process, some details are often not very clear and easy to interpret; sometimes this results in misdiagnosis, so medical professionals have become more and more interested in exploring new methods that can improve manual interpretation and analysis. Therefore, medical and engineering specialists have come to work closely together to develop new methods for providing a diagnosis as quickly and accurately as possible considering image processing and analysis as a post-imaging or pre-analysis step.

Ultrasound is one of the most used tools for acquiring medical images for screening and diagnosis in the field of obstetrics and gynecology. Ultrasound scanners capture reflections at the boundaries between and within tissues using the principle of pulse-echo imaging. Pulsed acoustic waves are transmitted and received by a transducer with frequencies for diagnostics ranging between 1 and 20 MHz. In general, higher frequencies produce better resolutions but penetration of the investigated tissues is lower, while lower frequencies produce greater depth of penetration but lower resolution.

Currently, ultrasound technology is of a high performance and provides a wide range of information, which gives the physician and the patient a high degree of confidence in the diagnosis. However, the problem of noise sources that cause many types of artifacts remains. As an example, the typical speckle artifacts in ultrasound images are caused by the fact that sound waves are highly distorted when traveling through the tissues, and these lead to distortion of the shape and texture of the structures and limit the detection of the obscure details in the medical images [1].

Consequently, in recent years, there has been a continuous increase in the number of methods supporting clinical decision making. More recent works are based on methods for the extraction and analysis of textural features of medical images and machine learning algorithms in order to classify them.

Textures of ultrasound images were analyzed by Park et al. [2] for studying the normal supraspinatus tendon and abnormal supraspinatus tendon with the histogram and gray level co-occurrence matrix. From the statistical results, the first order features provided an accuracy of 95% and GLCM of 85%.

The first order features were used to differentiate between normal and infarcted myocardial biostructures from ultrasound image textures. The results obtained by Moldovanu et al. [3] show that standard deviation and mean are the significant features. Moreover, FD is a solitary feature used for texture investigation and, in ultrasonography, FD characterizes malignant and benign endobronchial ultrasound nodes [4] or carotid plaque [5].

Another way of performing robust feature selection and classification of MR images, with direct applications to prostate cancer, is based on a combination of descriptive and inferential statistics, as described by Barone et al. [6]. Here the selection is made so that the set of features is the smallest one, which guarantees good predictive performance by using a correlation coefficient to evaluate any association between two observed features.

However, not all the features contain important information; therefore, in [7], a novel feature selection approach is presented, which creates a relevant predictive model used in classifying prostate lesions in MR images.

First order and texture features can also be classified with different artificial intelligent methods. Htay and Maung [8] proposed K-NN for early-stage breast cancer detection, Torheim et al. [9], in their study, analyzed the cervical cancers using texture analysis and support vector machines. The first order statistic features extracted from MR imagines were classified with K-NN, support vector machines and neural network, providing an accuracy of 97.37%.

The placenta is a transitory organ whose morphological and functional integrity is essential for fetal development. According to the normally expected aging process, ultrasound images of the placenta are different in respect with gestational age. These aspects are all included into the four very well-known grades of Grannum classification in which grade 0 stands for the uniform echogenicity of the placenta, whereas in grade III there are extensive calcium deposits in all placental tissue but mainly in the placental septic, giving the overall appearance of ring-like structures.

Echogenic foci in the placental tissue are seen in many ultrasound examinations, especially during the second or third trimester of pregnancy, as the calcium progressively deposits [10], and these changes are usually considered as normal aspects of aging and maturation [11].

Placental calcifications are reported differently in the literature, but most of the studies showed that more than 50% of the placentas have some degree of calcifications, whereas more than 18% have extensive calcium deposits [12]. In a study published by Miller et al. in 1988, placental calcifications were seen in more than 39% of pregnancies at term [13].

In most cases, minor placental calcifications can be seen as the pregnancy progresses. However, cigarette smoking during pregnancy, certain medications or vitamin supplements as well as pregnancy associated pathology such as placental abruption or pregnancy associated hypertension are more often correlated with early and more extensive calcifications [14].

**Figure 1:**
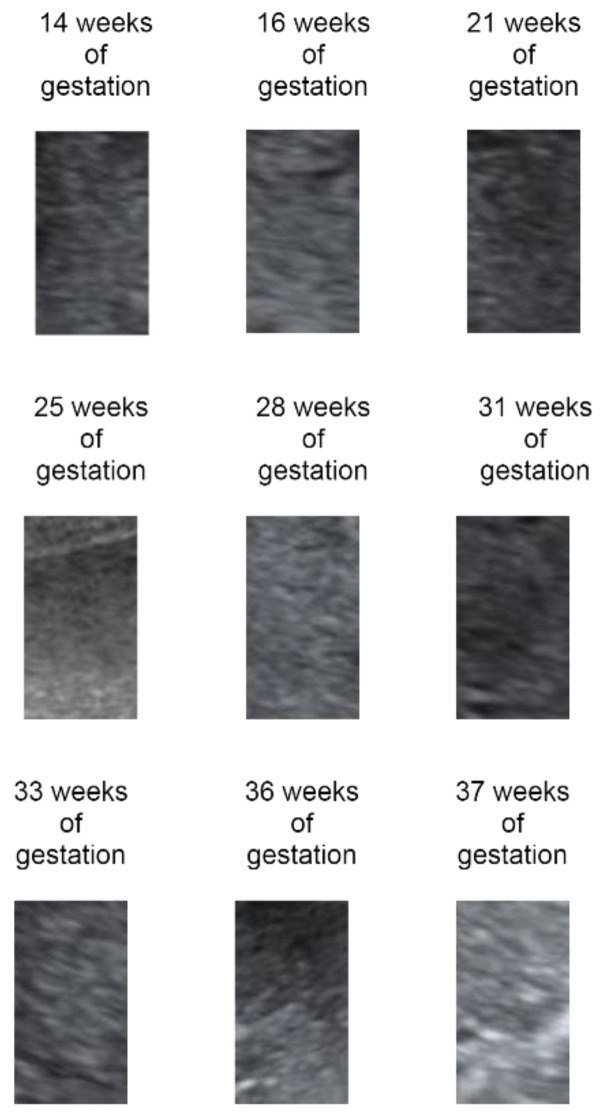
A representative example of placental evolution during pregnancy.

A representative example of a placental aging process can be seen in Figure 1 where several regions of interest (ROI) from various pregnancy ages are shown.

The prevalence of preterm placental calcifications, whether early, before 32 weeks, or late, between 32 and 36 weeks of gestation, is reported with different numbers in many studies from the literature.

In a study published in 2005 by McKenna et al., the prevalence of placental calcifications at 36 weeks of gestation was only 3.8% [15].

In other studies, the prevalence of placental calcifications was 9% before 28 weeks of gestation [16], 15% between 34 and 36 weeks [17] and 23.7% between 31 and 34 weeks of gestation [18].

The significance of preterm placental calcifications is not fully understood. Moreover, the reports from the literature show diverging data. Whether some studies show no interference between placental calcifications and normal fetal growth and overall outcome, there are reports that strongly correlate early preterm calcifications with intrauterine growth restriction [15,18], low birth weight [15,17,18,19], low Apgar score [17], neonatal death [10] and pregnancy-induced hypertension [15,20]. Moreover, postpartum hemorrhages seem to be more frequent in pregnancies with preterm placental calcifications [10].

In this study, a method for analyzing the placenta structure based on the first, second and fractal dimension is proposed. The original image is divided into ROIs from which the proposed features are extracted. As well as the first and second features, the fractal dimension is extracted to follow the fine structure of microcalcifications. All features were tested with the t-test, taking into consideration the significance, and then with the K-NN algorithm; the classification accuracy was generated. To highlight the microcalcifications area in the preprocessing step an unsharp filter was applied.

The rest of this paper has the following structure: Section 2 presents the materials and methods proposed in the context of improving the detectability of microcalcifications in the placenta during pregnancy using textural features and the K-Nearest Neighbors algorithm. The results of the study, performance comparison with results from the SVM classifier and a brief discussion are presented in Section 3. And Section 4 draws the conclusions of the paper.

## 2. Materials and Methods

Medical images can be seen as processes in which the characteristics of the tissue are translated into a shade of gray or RGB image.

The information contained in an image and perceived by the human eye through features such as brightness, color, texture or edges may be affected by the sensitivity and resolution of the acquisition equipment, noise and artifacts added over the useful signal.

Due to the nature of the technology used, even if it is currently very performant, most of the acquired medical images are affected by various disturbances that lead to a diminished contrast and visibility, especially in the case of fine details.

Improving the details of an image highlights its fine details that have been blurred or diminished due to errors occurring as a result of the acquisition method. However, a disadvantage of the contrast enhancement method is that the noise can also be accentuated [21].

For this reason, the main contribution of this paper is the analysis of the placental structure in areas where the microcalcifications are most likely to appear so that a correct classification and selection of areas with microcalcifications can be made.

**Figure 2:**
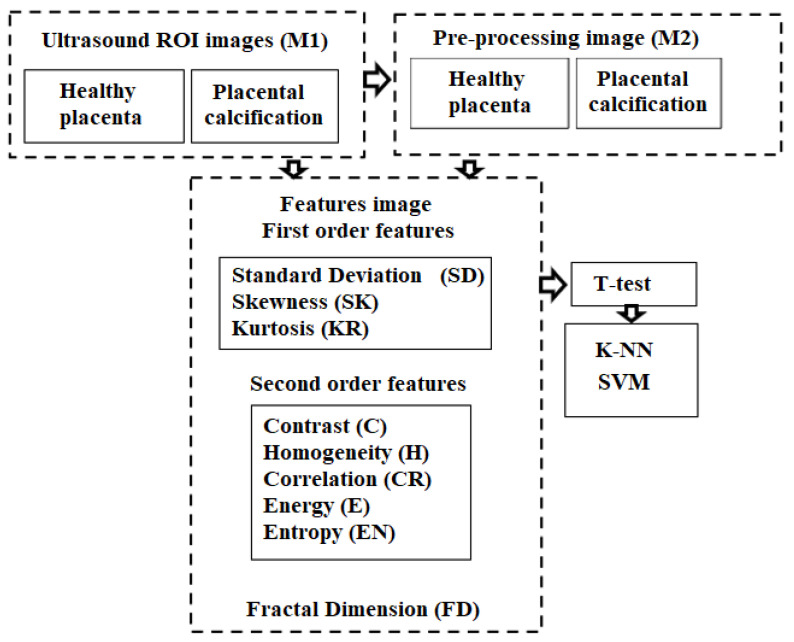
Algorithm diagram.

The paper proposes a new technique to improve the detectability of details in predefined regions of interest in order to achieve a better contrast between different colors by combining filtering methods, existing filtering techniques of the placental structure analysis using textural features and K-Nearest Neighbors algorithm. For comparing classification results of the K-NN algorithm, a support vector machine (SVM) classifier is also applied due to its outstanding generalization capability [22] and reputation to achieve high accuracy with less computational complexity for many practical problems from a medical imaging field [23]. The algorithm diagram can be seen in Figure 2.

The proposed methodology was applied to raw data, marked as M1 method and repeated for preprocessed images with an unsharp filter, marked as M2. For both M1 and M2, the regions of interest were automatically generated by imposing a given ROI size, and their selection and validation relied on the specialist human observer according to the specific task.

Firstly, the approach required the implementation of a filtering method especially suited for the detection of placental microcalcification; secondly, in order to increase the accuracy of medical evaluation, a statistical textural features and signification checking and performance analysis via different classification paradigms was performed on both M1 and M2.

### 2.1. Preprocessing Operations

#### 2.1.1. Contrast Enhancement

Increasing the contrast of the image aims at improving the visual perception of the contours of objects because it is well known that human perception is sensitive to edges and fine details. In general, this goal can be achieved by changing the pixel values on both sides of a common border. The border can be seen as a transition from white to black (from one color to another). Therefore, improving the contrast should produce a rapid transition from white to black, which eventually leads to a clearer picture. Instead, a gradual transition from white to black through multiple levels of gray leads to blurry images and blurry details.

In this context, we implemented a filtering method based on a classical approach of image processing. This method consists of two steps: the first one is filtering the image with a high-pass filter because information about small details is contained in the high frequencies; the second one consists of obtaining a controlled, enhanced contrast by adding to the original image a weighted filtered image [21,24].

**Figure 3:**
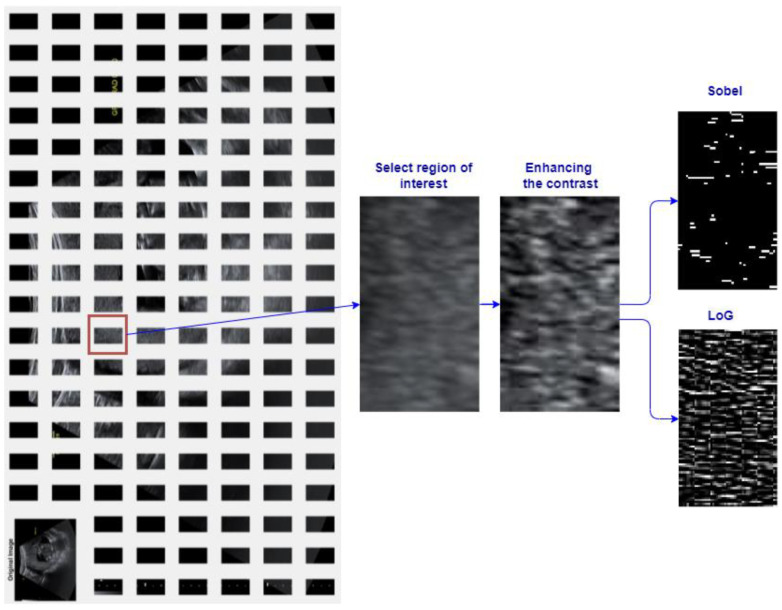
Illustration of regions of interest (ROI) and preprocessing operations for contrast and edge enhancement.

The method was applied on cropped ROI of the images containing the microcalcifications, which were validated by a human expert. The result of applying the filtering method to increase the visibility and detectability of microcalcifications in a 14 weeks of gestation placenta are presented in Figure 3.

#### 2.1.2. Edge Enhancement

Microcalcifications are fine shapes, white specks, sometimes like grains of salt. In computer vision, fractal dimension is a tool extensively used for characterizing roughness and self-similarity.

To analyze microcalcifications, the box counting method (BC) is proposed for the 2D images as it provides values in the range of 1 and 2.

Before applying the BC, the edges of the gray level image were detected with first and second derivative filter. From the first category, the Sobel filter and the second Laplacian of Gaussian (Log) filter were chosen, and its preprocessing results are shown in Figure 3.

The FD computed with BC after the Sobel filter was applied as noted with FD_I and with FD_II after LoG detected the edges.

### 2.2. Texture Analysis

#### 2.2.1. First Order Features

First order features are computed from the histogram image. In this study, standard deviation, skewness and kurtosis were considered.

Standard deviation measures the distribution of intensity values about the mean.

(1) $$SD=\sqrt{\overset{L}{\underset{i=1}{\sum }}\left(i-M\right)P\left(i\right)}$$

Skewness measures the unequal distribution or asymmetry of intensity values of histograms about the mean value.

(2) $$SK=\frac{1}{{\left(SD\right)}^{3}}\overset{L}{\underset{i=1}{\sum }}{\left(i-M\right)}^{3}P\left(i\right)$$

The Kurtosis calculates the average amount of the peak of intensity distribution values (sharpness).

(3) $$KR=\frac{1}{{\left(SD\right)}^{4}}\overset{L}{\underset{i=1}{\sum }}{\left(i-M\right)}^{4}P\left(i\right)$$

where *P*(*i*) is the probability of gray levels and *M* is the average of the intensity values obtained from the bins of histograms and *L* belongs the range 0 to 255 [25].

#### 2.2.2. Second Order Features

Gray level co-occurrence matrix (GLCM) is a main tool in image texture analysis, moreover a statistical method that analyzes image texture structure of gray levels’ occurrence in comparison with other gray levels. For a GLCM with *N* × *M* size, the next standard features as contrast (C), homogeneity (H), correlation (CR), energy (E) and entropy (EN) are computed from a normalized co-occurrence matrix.

(4) $$C=\overset{L-1}{\underset{i=0}{\sum }}\overset{L-1}{\underset{j=0}{\sum }}{\left(i-j\right)}^{2}N_{p}\left(i,j,\delta ,\theta \right)$$

(5) $$H=\overset{L-1}{\underset{i=0}{\sum }}\overset{L-1}{\underset{j=0}{\sum }}\frac{N_{p}\left(i,j,\delta ,\theta \right)}{1+\left|i-j\right|}$$

(6) $$CR=\overset{L-1}{\underset{i=0}{\sum }}\overset{L-1}{\underset{j=0}{\sum }}\frac{\left(i-\mu_{i}\right)\left(j-\mu_{j}\right)N_{p}\left(i,j,\delta ,\theta \right)}{\sigma_{i}\sigma_{j}}$$

(7) $$E=\overset{L-1}{\underset{i=0}{\sum }}\overset{L-1}{\underset{j=0}{\sum }}N_{p}^{2}\left(i,j,\delta ,\theta \right)$$

(8) $$EN=\overset{L-1}{\underset{i=0}{\sum }}\overset{L-1}{\underset{j=0}{\sum }}N_{p}\left(i,j\right){log}_{2}N_{p}\left(i,j,\delta ,\theta \right)$$

where *L* = 256 represents the grey level, $N_{p}\left(i,\;j,\;\delta ,\;\theta \right)$, the probability that two grey levels occur at the same time at δ and at angle $\theta \in \left\{0^{\circ },45^{\circ },90^{\circ },135^{\circ }\right\}$. $\mu_{i},\;\mu_{j},\;\sigma_{i}$ and $\sigma_{j}$ are the mean and standard deviation of $N_{p}\left(i,\;j,\;\delta ,\;\theta \right)$ [26].

#### 2.2.3. Fractal Dimension and First and Second Derivative Filter

Fractal dimension (FD) is based on the concept of self-similarity. In image processing FD is defined as

(9) $$FD=\underset{r\to \infty }{lim}\frac{log\left(N_{r}\right)}{log\left(\frac{1}{r}\right)}$$

where $N_{r}$ is the least number of distinct copies of the 2D image in at the scale *r* [27].

The box counting method was applied on the binary image obtained with first and second derivative filter. From the first order derivative filter category was used Sobel filter and from the second order derivative filter category, the Laplacian Gaussian filter was applied.

### 2.3. Statistical Analysis and Classification

The statistical significance of first, second and FD features was performed using a t-test. Thus, the null hypothesis (all actual mean values are the same) is tested for each feature extracted from healthy and calcified regions of interest because it is expected that the features have significantly different values for the two cases considered. Three first order statistics, 5 GLCM features and two FD features (FD_I and FD_II) with a *p*-value under 0.05 are selected to be the inputs of the classification algorithm.

In this study, we used a K-NN to classify texture features and accuracy is used to evaluate performance. K-NN algorithm is a popular and powerful machine learning algorithm, and widely used in binary classification problems.

On the data set consisting of first and second features, and fractal dimension, the K-NN algorithm is applied. The resulting performance of the prediction is obtained via the confusion matrix (CM). The accuracy (see Equation (10)) is computed based on the following values provided by the CM [28]: true positive (TP), true negative (TN), false positive (FP), false negative (FN).

(10) $$Accuracy=\frac{TP+TN}{TP+FP+TN+FN}\left(\%\right)$$

### 2.4. Practical Implementation

The results presented in this study consist of 96 regions of interest from images corresponding to different weeks of pregnancy, mainly before the 32 weeks of gestation. The images were obtained with the informed consent of patients.

The images acquisition was realized with Voluson E6 machine (General Electric Company, Boston, MA, USA) equipped with a 2–8 MHz transabdominal GE RAB6-D transducer (Jaken Medical Inc., Chino, CA, USA). The resolution of images was by 960 × 720.

Statistical analyses, classification and image processing were performed using MATLAB R2020b with Image processing and statistic toolboxes (The MathWorks, Inc., Natick, MA, USA). The hardware used was a computer with the following specifications: Inter (R) Core (TM) i7-8550U CPU @ 1.80 GHz (Intel Corporation, Santa Clara, CA, USA); Memory (RAM) 8 GB DDR4 Samsung, Pyeongtaek, Korea).

## 3. Results and Discussions

In this paper, we developed a quantitative ultrasound texture analysis to accurately detect microcalcifications in the placenta during pregnancy. After the preprocessing stage is completed, the next stage is feature extraction and analysis for the two classes, healthy and calcified placenta. The features for our study are selected according to their correlation to calcified placenta. From the 10 texture features analyzed with the t-test, the results are all salient (*p* < 0.05); thus, all are further considered to be inputs for the K-NN algorithm.

In Table 1, the *p*-value resulted from the t-test for each feature extracted from the raw images (M1) and our proposed method (M2) can be seen.

Furthermore, K-NN is used to classify the images in two classes: healthy and calcified placenta. For this purpose, the data are divided into two sets: 80% training data and 20% test data. In the next step, for k-fold cross validation, the training data are split into k = 5 subsets. The classifier is trained and validated by k separate times: with a training group consisting of k-1 subsets (4 subsets × 16%) and with a remaining subset (1 subset × 16%) for cross validation. The accuracy for each k experiment is computed. However, the performance of the classifier is tested with unseen data from the validation set (20%). The best accuracy of the algorithm is achieved when the number of neighbors is K = 3.

To differentiate between healthy and calcified placenta a combination of features is initially considered for both methods, M1 and M2. In this case, the accuracy of the classifiers obtained with K-NN and 5-fold cross validation procedure is lower than 75%. The low performance results are:

(i) first order features and second order features, M1 (68.3%), M2 (68.8%);
(ii) first order features, second order features and FD, M1 (70.1%), M2 (71.1%);
(iii) first order features and FD, M1 (73.4%), M2 (73.2%);
(iv) second order features and FD; however, their clustering leads to a decrease in the accuracy, M1 (68.1%), M2 (69.1%).

Consequently, analyzing the textural features individually leads to better performance. The only downside is that for each feature, a separate classifier is required to be trained. However, our study’s purpose is not negatively impacted by this.

**Figure 4:**
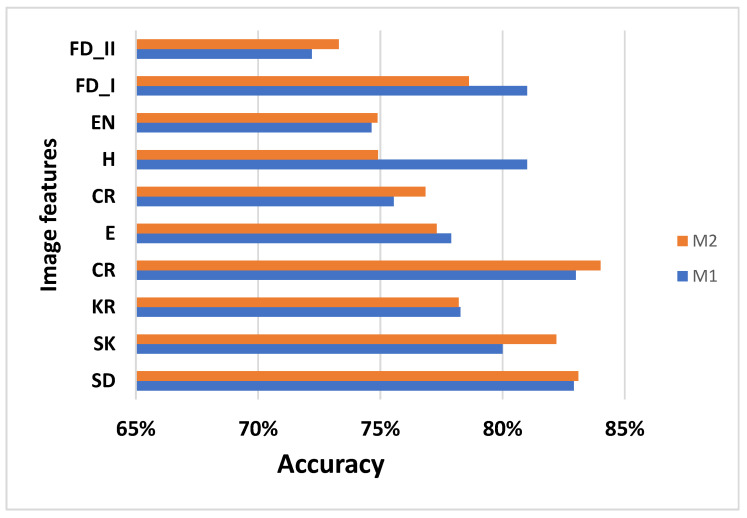
Performances for M1 and M2 methods obtained with K-NN.

The results obtained are presented in Figure 4 and show that the proposed method (M2) provides good performance and high accuracy in comparison with M1 (raw images). This is demonstrated by the GLCM features from M2: CR obtains high accuracy (84.01%) and SD provides 83.01% accuracy.

Unfortunately, after applying the microcalcifications’ enhancement in the preprocessing stage, FD_I and FD_II become low-level features.

As the paper shows a classification problem, a support vector machine classifier (SVM) is applied further to check if the accuracy of both methods (M1 and M2) can be improved. After testing several kernel functions, the polynomial kernel function proved to be the most appropriate for the SVM.

**Figure 5:**
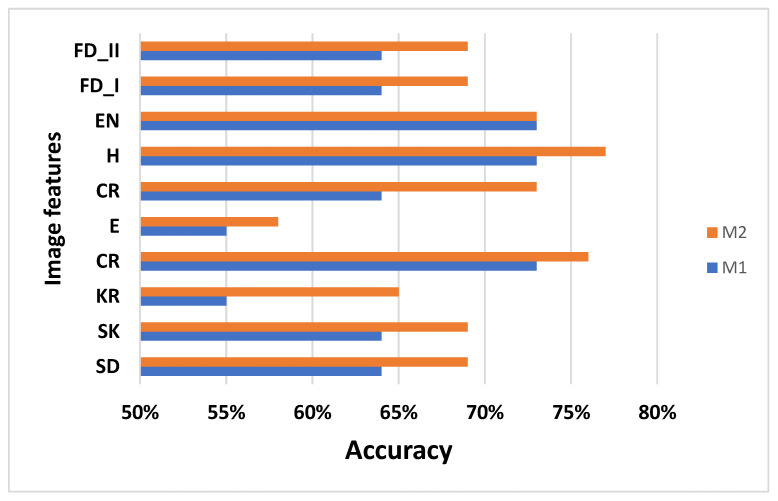
Performances for M1 and M2 methods obtained with SVM.

The results from Figure 5, show that the accuracy of M1 and M2 are not improved with the SVM algorithm; moreover, they present lower performances in comparison with the values from the K-NN algorithm. This is observed from the GLCM features from M2: CR has 76% accuracy and SD obtains only 69% accuracy.

All values of accuracy are obtained by evaluating the SVM classifier with a test set, generated by splitting the images in two categories: 80% for training and 20% for testing.

This is a pioneering study of the texture and classification of placenta microcalcifications; therefore, there are no comparable studies from the scientific literature. The proposed image detecting and classification method yield very good results, especially when analyzing ultrasound images obtained before 32 weeks of gestation.

By using it, it is possible to have a fast diagnosis tool and also to better track the evolution of preterm placental microcalcifications; in principle, small microcalcifications that are difficult to identify in the unprocessed image.

## 4. Conclusions

According to many studies from the literature, preterm placental calcifications could be associated with poor maternal and fetal outcome. That is why placental calcifications, especially when their presence is noted early in the pregnancy, should be treated with extreme caution and regarded as a potential placental dysfunction instead of a normal aging process.

As a result of our study, an image enhancement technique combined with techniques of placental structure analysis could be very useful in the detection of early placental microcalcifications.

Classification plays an important role in this, improving the separation into healthy and calcified placenta. This process ensures the early detection of preterm placental calcifications, which, in turn, could improve the overall fetal and maternal outcome through a closer surveillance and proper counselling of the pregnant woman.

In this paper, two different classifiers (K-NN and SVM) were investigated and compared to find out which model would be suitable for our dataset and salient features data. The accuracy of the classifiers obtained when using the K-NN, with a 5-fold cross validation, outperforms the SVM results in our case study. However, is important to note that hyperparameter optimizations and selected features play a critical role in obtaining a good classification. Different algorithms, based on nature-inspired approaches [29], such as the convolutional neural network (CNN), that are capable of automatically extracting features from raw data without prior knowledge about the dataset labels can be examined in future work. Additionally, we plan to investigate unsupervised segmentation techniques [30] to overcome the effort needed to be spent on extracting and selecting classification features.

## Figures and Tables

**Table 1:** The *p*-value from t-test computed for first, second and FD features.

	SD	SK	KR	C	H	CR	E	EN	FD_1	FD_2
M1	0.012	0.043	0.041	0.033	<0.001	0.032	0.038	<0.001	0.045	0.048
M2	<0.001	0.035	0.04	0.008	0.002	0.030	0.022	<0.001	0.043	0.043

## Data Availability

Not applicable.
